# Tumour-microenvironmental blood flow determines a metabolomic signature identifying lysophospholipids and resolvin D as biomarkers in endometrial cancer patients

**DOI:** 10.18632/oncotarget.22558

**Published:** 2017-11-20

**Authors:** Núria Eritja, Mariona Jové, Kristine Eldevik Fasmer, Sònia Gatius, Manuel Portero-Otin, Jone Trovik, Camilla Krakstad, Joaquim Sol, Reinald Pamplona, Ingfrid S. Haldorsen, Xavier Matias-Guiu

**Affiliations:** ^1^ Department of Pathology and Molecular Genetics/Oncologic Pathology Group, Arnau de Vilanova University Hospital, University of Lleida, IRBLleida, Lleida, Spain; ^2^ Centro de Investigación Biomédica en Red de Oncología (CIBERONC), Madrid, Spain; ^3^ Department of Experimental Medicine, University of Lleida, IRBLleida, Lleida, Spain; ^4^ Department of Radiology, Haukeland University Hospital, Bergen, Norway; ^5^ Department of Clinical Medicine, University of Bergen, Bergen, Norway; ^6^ Department of Obstetrics and Gynecology, Haukeland University Hospital, Bergen, Norway; ^7^ Department of Clinical Science, Center for Cancer Biomarkers, University of Bergen, Bergen, Norway; ^8^ Department of Pathology, University Hospital of Bellvitge, Bellvitge Biomedical Research Institute (IDIBELL), L’Hospitalet de Llobregat, Catalonia, Spain

**Keywords:** endometrial cancer, DCE-MRI, blood flow, metabolomic analysis

## Abstract

**Purpose:**

We aimed to study the potential influence of tumour blood flow –obtained from dynamic contrast-enhanced magnetic resonance imaging (DCE-MRI)- in the metabolomic profiles of endometrial tumours.

**Methods:**

Liquid chromatography coupled to mass spectrometry established the metabolomic profile of endometrial cancer lesions exhibiting high (n=12) or low (n=14) tumour blood flow at DCE-MRI. Univariate and multivariate statistics (ortho-PLS-DA, a random forest (RF) classifier and hierarchical clustering) and receiver operating characteristic (ROC) curves were used to establish a panel for potentially discriminating tumours with high versus low blood flow.

**Results:**

Tumour blood flow is associated with specific metabolomic signatures. Ortho-PLS-DA and RF classifier resulted in well-defined clusters with an out-of-bag error lower than 8%. We found 28 statistically significant molecules (False Discovery Rate corrected p<0.05). Based on exact mass, retention time and isotopic distribution we identified 9 molecules including resolvin D and specific lysophospholipids associated with blood flow, and hence with a potentially regulatory role relevant in endometrial cancer.

**Conclusions:**

Tumour flow parameters at DCE-MRI quantifying vascular tumour characteristics are reflected in corresponding metabolomics signatures and highlight disease mechanisms that may be targetable by novel therapies.

## INTRODUCTION

Endometrial cancer (EC) is the most common gynaecologic malignancy in the high-income countries, and its incidence has increased by 21% since 2008 [1]. Presently, the treatment and prognosis are influenced by a subset of parameters based on molecular markers and the microscopic appearance of the tumours. Although approximately 75% of patients are primary curable by surgery and adjuvant treatment, 15-20% of these tumours will recur, with an aggressive and therapy-resistant behavior [2]. Several events (including genetic alterations and tumour microenvironment factors) have been reported to be activated or co-existing in aggressive endometrial cancer [3].

Angiogenesis is an essential mechanism for tumour growth, invasion and metastatic spread in many cancers types [4]. Moreover, tumour-associated vessels are frequently structurally and functionally abnormal, exhibiting delayed maturation and increased permeability [5].

In EC, increased microvascular proliferation correlates with a more aggressive phenotype [6-8]. Furthermore, high microvascular proliferation is associated with lower tumour blood flow (F_b_) based on dynamic contrast-enhanced magnetic resonance imaging (DCE-MRI), and low tumour F_b_ is observed in EC patients with reduced recurrence /progression-free survival [6, 9]. Altogether, is consistent with the hypothesis that vascular proliferation with coexisting disorganized angiogenesis promotes tumour progression and metastatic spread in EC [6].

Cancer was primarily considered a proliferative disorder; however, altered cell metabolism has emerged as a “hallmark of cancer”, since growing tumour cells rewire their metabolic program to meet the biosynthetic and bioenergetic demands of constant cell growth and phenotype changes. Metabolomics analysis provides direct evidences of biochemical context-dependent changes that can be correlated with the pathologic state of a cell, tissue or organ [10]. Therefore, metabolomics has been applied to reveal new mechanisms in cancer development and has identified specific biomarkers relevant for diagnosis and prognosis in various cancers.

The present study explores the different metabolomic tumour signatures potentially characterizing EC lesions exhibiting high versus low tumour blood flow at DCE-MRI. The identification of high versus low F_b_ metabolic biomarkers may highlight preoperative functional tumour characteristics with potential relevance for preoperative risk assessment and for identification of EC patients with expected poor outcome.

## RESULTS

The main objective of this work was to determine metabolomics differences based on tumour F_b_ using a non-targeted metabolomics approach. We employed multivariate statistics to study changes in whole sample metabolome both in unsupervised-principal component (PCA) and hierarchical clustering analysis- and in supervised approaches-partial least discriminate analysis (PLS-DA), ortho-PLS-DA and Random Forest (RF) [11].

Patients had a mean age of 68 years and 20/26 (77%) of tumours had endometrioid histology (Table 1). In total 17/26 (66%) were classified as early stage (FIGO 1: Federation International of Gynecology and Obstetrics), and received no adjuvant therapy in accordance with national guidelines. 4/6 (67%) of non endometrioid histological subtype patients received adjuvant chemotherapy.

**Table 1:** Clinicopathological patient characteristics (n = 26) of women treated for endometrial cancer and evaluated by enhanced contrast MRI and metabolomics

**Age; mean (range) years**	68 (41-93)
Postmenopausal, n (%)	23 (89)
Lymph node sampling performed, n (%)	21 (81)
Adjuvant therapy received, n (%)	9 (35)
**FIGO stage**^a^**, n (%)**	
IA	10 (39)
IB	7 (27)
II	4 (15)
III	4 (15)
IV	1 (4)
**Histologic differentiation, n (%)**	
Grade 1	5 (19)
Grade 2	5 (19)
Grade 3	16 (62)
**Histologic subtype, n (%)**	
Endometrioid	20 (77)
Clear cell	1 (4)
Serous	3 (11)
Carcinosarcoma	2 (8)

^a^According to International Federation of Gynaecology and Obstetrics (FIGO) 2009 staging criteria (ref)

**Figure 1:**
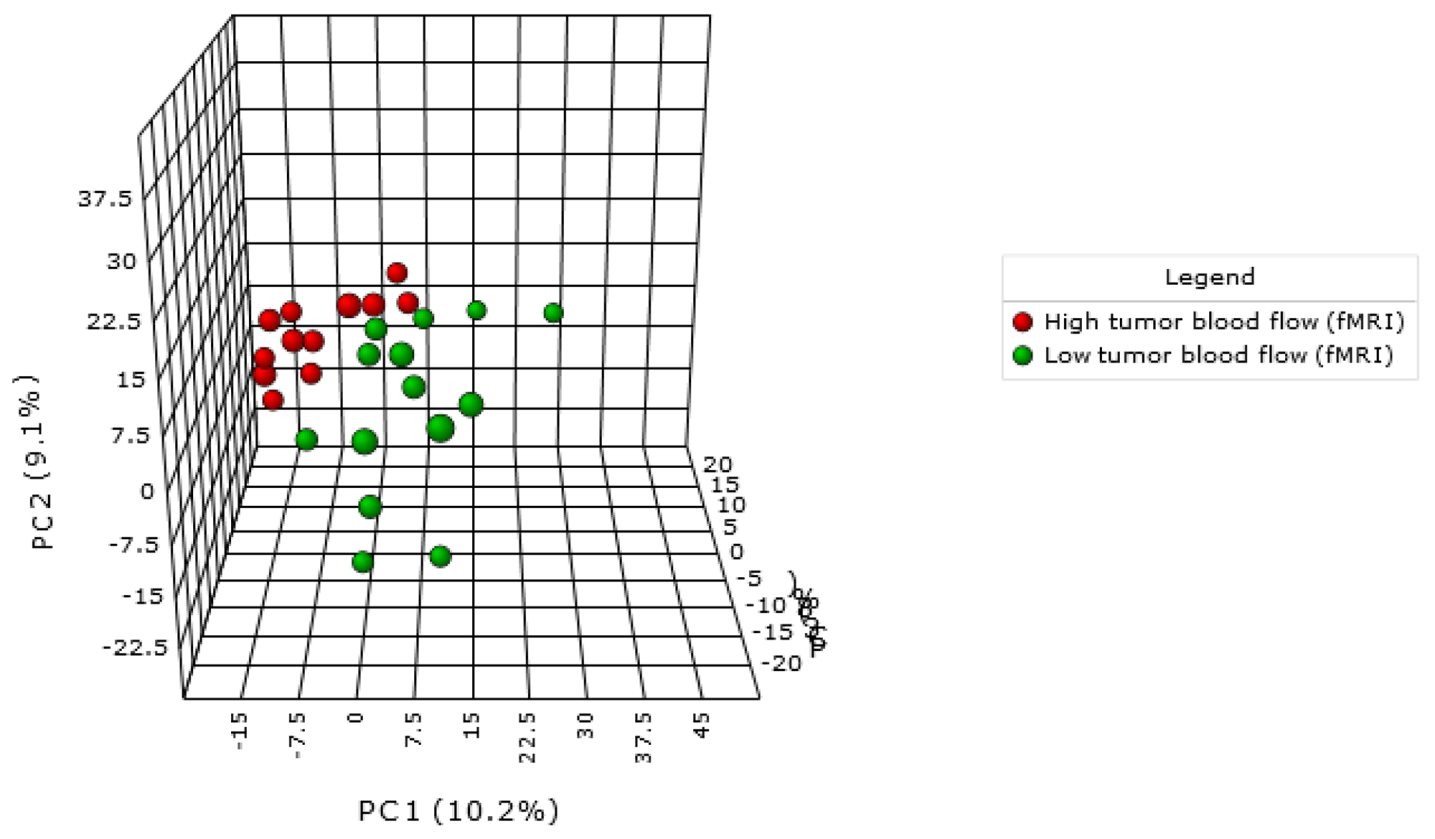
PCA demonstrate a metabolomic-specific signature depending on tumour blood flow in endometrial cancer patients Red spots represent high tumour blood flow and green spots represent low tumour blood flow as determined by DCE-MRI.

**Figure 2:**
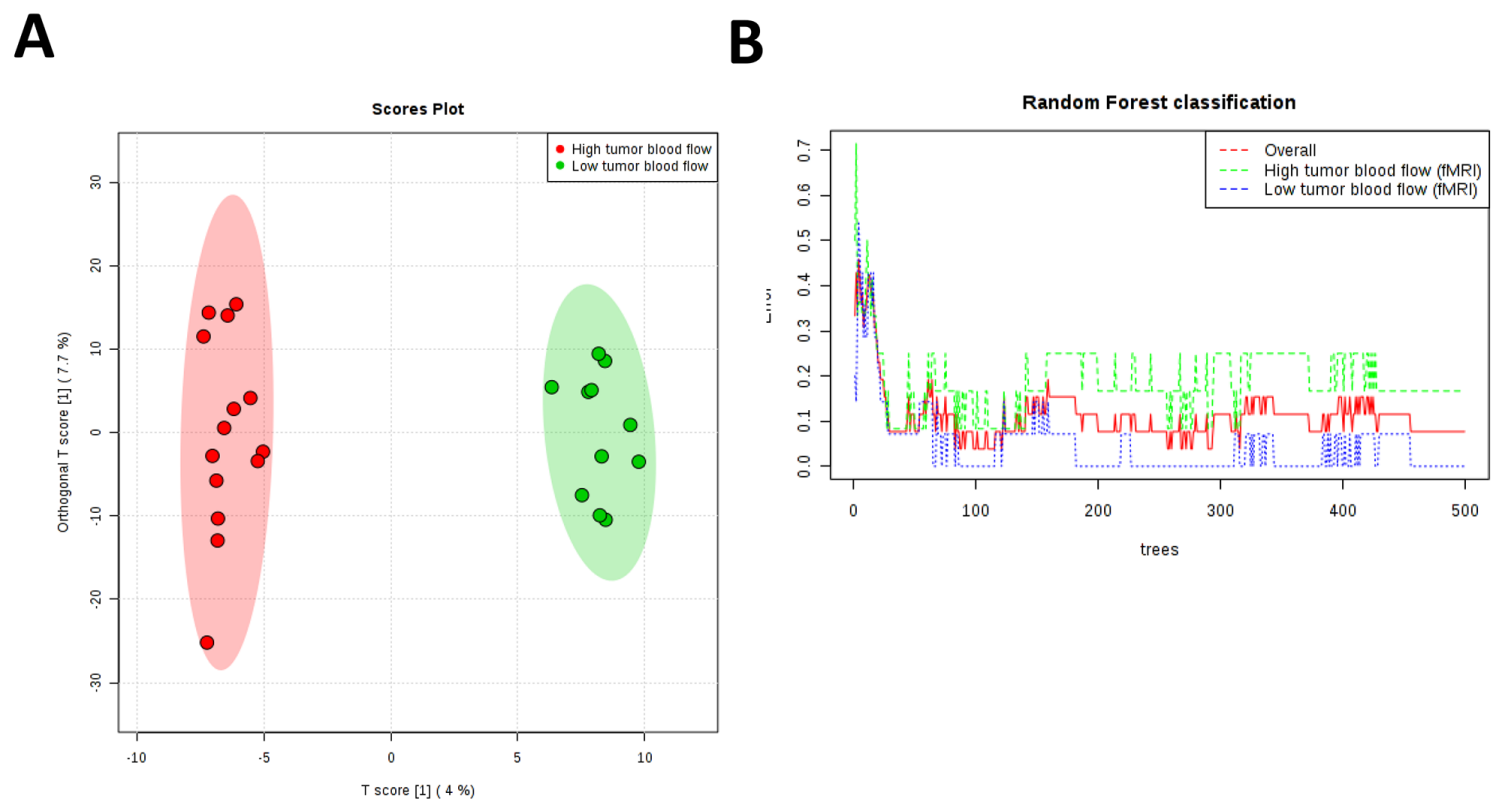
**(A)** Ortho-PLSDA algorithm using all metabolites found in samples from endometrial cancer tumours is able to discriminate groups with an observed Q2 of 0. 656 and observed R2Y of 0.985. Red spots represent high tumour blood flow patients and green spots represent low tumour blood flow as determined by DCE- MRI. **(B)** RF classification shows and overall classification error of 0.07.

In the present work PCA analysis was chosen as an unsupervised method (Figure 1). This analysis showed that lesions with high tumour F_b_ clustered better than lesions exhibiting low tumour blood flow. Once we discarded the presence of outliers and checked the technical quality of the technique we applied ortho-PLSDA as a specific tool for clustering (Figure 2A). This analysis resulted in two well-defined clusters (Q2=0.656 and R2Y =0.985). RF analysis reinforced these results showing an out of bag error of 0.07 (Figure 2B). Because of endometrioid and non-endometrioid tumours are different EC subtypes we also performed statistics excluding non-endometrioid EC patients obtaining similar results (Supplementary Figure 1A-1C). Specifically multivariate statistics show a good culsterization according to high and low tumour F_b_ (ortho-PLSDA parameters: Q2 =0.553 and R2Y =0.979, random forest out of bag error = 0.1).

**Figure 3:**
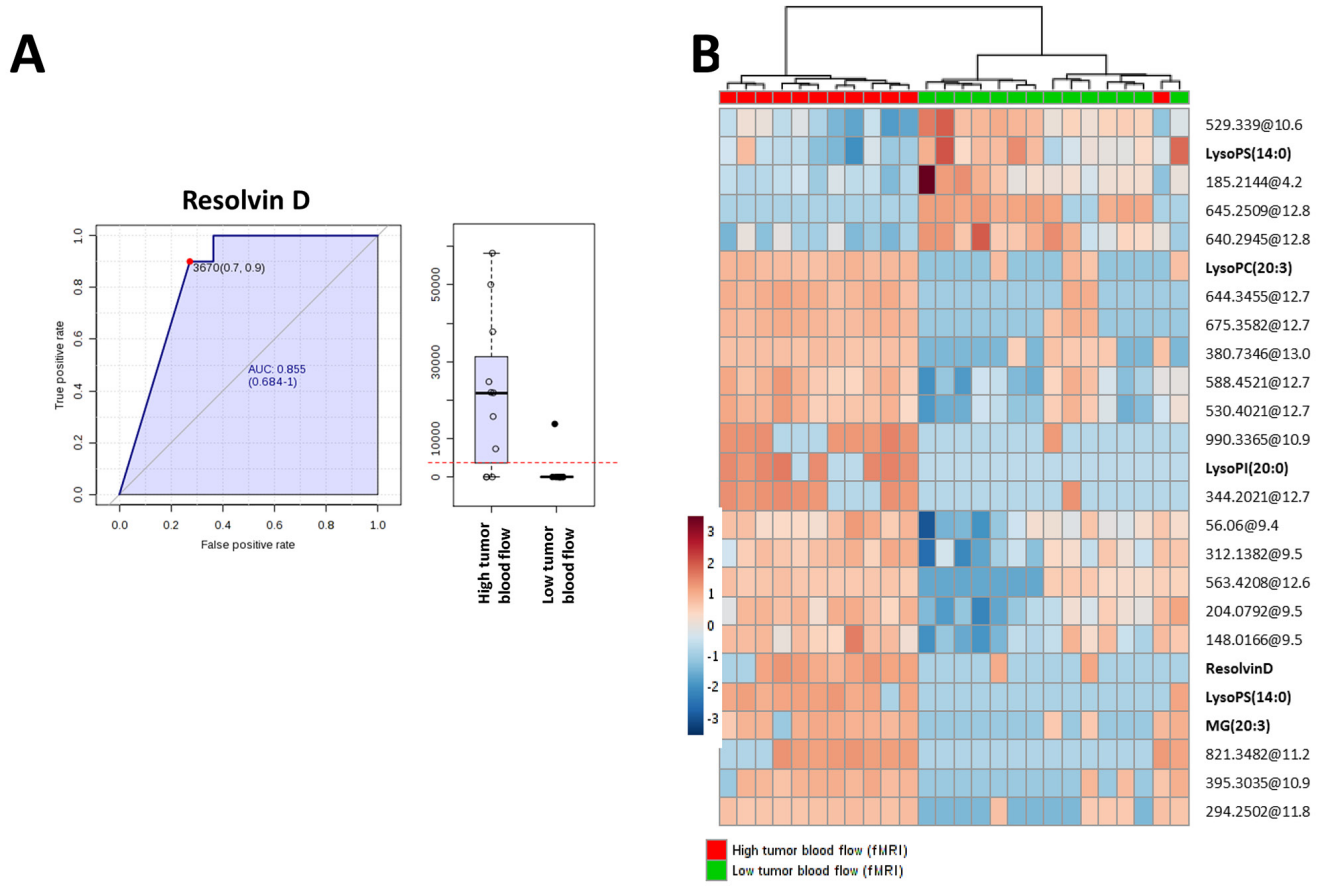
**(A)** ROC curve of tumour resolvin D levels. **(B)** Hierarchical clustering analyses using 25 most statistical significant metabolites significantly different between endometrial cancer tumours with high versus low blood flow (Student’s T-Test, Benjamini Hochberg False Discovery Rate, p<0.05). Unknown identities are represented as exact mass@retention time.

The analysis revealed 28 statistically significant molecules (Table 2). The annotation process (using exact mass, retention time and isotopic distribution) disclosed several lipids belonging to phospholipid, glycerolypid and sphinogolipid families. Specifically, four lysophosphatidylcholines were up-regulated in tumours with high F_b_. Moreover, resolvin D was identified as a potential tumour F_b_ biomarker. The ROC curve of resolvin D (AUC= 0.855, specificity= 0.9 and sensitivity = 0.7) confirmed the robustness of this metabolite to predict high tumour F_b_ (Figure 3A). To further study the predictive value of the metabolites found, we applied ROC curves using groups of metabolites (5, 10, 15, 25, 50 and 100) with lowest p-value obtained from T-Test between EC tumours with high versus low F_b_ observing a higher sensitivity and specificity when only the statistical significant metabolites (T-Test, Benjamini Hochberg False Discovery Rate, p<0.05) with a potential identity were used (Supplementary Figure 2). Using these metabolites we could reach a higher predictive value than using only Resolvin D1. Finally, we applied hierarchical clustering analyses using the 25 most statistically significant metabolites and an almost perfect separation between groups was observed (Figure 3B).

**Table 2:** Metabolites from endometrial cancersignificantly different expressed in tumors with high versus low blood flow (Fb) determined by dynamic contrast enhanced (DCE)-MRI blood flow (Student’s T-Test, p<0.05)

Potential ID (exact mass)	p (Corr)	Regulation (H vs L)	FC	Mass	Retention Time
Unknown	0.001717	down	-3140.98	645.2509	12.776092
Unknown	0.002955	down	-1.55451	529.339	10.614731
Unknown	0.002955	down	-2.36942	640.2945	12.776231
LysoPS(14:0)	0.002955	up	1313.153	469.2301	11.289364
Unknown	0.002955	up	2939.315	644.3455	12.713154
MG(20:3)	0.005209	up	2786.49	380.2894	11.154073
LysoPI(20:0)	0.00663	up	451.2966	628.3694	12.711375
LysoPC(18:1)	0.0079	up	7360.114	521.3482	11.1267
Unknown	0.0079	up	744.7858	675.3582	12.709785
Unknown	0.0079	up	784.6694	380.7346	13.018824
Unknown	0.008335	up	3.061549	588.4521	12.71631
Unknown	0.008335	up	1489.749	395.3035	10.927358
Unknown	0.009024	up	4.129054	530.4012	12.716731
Unknown	0.010229	up	584.1685	294.2502	11.812234
Unknown	0.017847	down	-1.76119	497.3935	5.910731
LysoPC(20:3)	0.027094	up	1097.7	545.3473	10.9397335
Unknown	0.029734	down	-1.53841	185.2144	4.237846
Resolvin D	0.03647	up	480.5512	376.2332	10.606182
Unknown	0.03647	down	-135.16	639.5252	12.372849
PC(42:2)	0.03647	down	-251.465	869.6867	13.405749
Unknown	0.03647	down	-175.365	915.7157	13.490999
Unknown	0.03647	up	319.0776	563.4208	12.652739
Unknown	0.03647	up	275.0069	344.2021	12.714
Unknown	0.03647	down	-297.762	890.7981	14.891124
NeuAcalpha2-3Galbeta-Cer(d34:1)	0.03647	up	274.8682	990.6635	10.921999
Unknown	0.03647	up	383.5934	410.2134	11.286181
Unknown	0.04131	up	861.9869	901.5447	13.720384
PI-Cer(t46:0(2OH))	0.044655	up	223.0672	981.7524	12.743363

## DISCUSSION

Advanced DCE-MRI techniques enable visualization and quantification of functional tumour characteristics *in vivo*. Previous studies revealed that in EC, low tumour F_b_ accompanied by increased capillary leakage and high microvascular proliferation is associated with reduced recurrence/progression free survival [6, 9]. These associations are somewhat puzzling, but may be related to the fact that tumoural angiogenesis generates vessels that in spite of being abundant, are heterogenous and rough making them altogether dysfunctional [12]. Further, the negative impact of low tumour F_b_ and disorganized angiogenesis have suggested tumour hypoxia promotes tumour progression and metastatic spread in endometrial carcinomas [13].

Based on these premises we designed an experiment to evaluate potential changes in tissue metabolome according to whether the tumours preoperatively exhibited high- or low F_b_.

Previous studies have suggested a specific metabolomics signature of EC compared to normal tissue [14], but the present results indicate that there is a specific metabolome associated with tumour F_b_, suggesting important changes in tumour metabolism which could modulate EC progression and angiogenesis related resistance to treatment [15]. Among all statistically different molecules found, we detected lipid species belonging to different families including phospholipids and, specifically, lysophospholipids (LPL). LPL were first discovered as constituents of cell membranes; however, today we know that they can also act as signalling molecules, playing important roles in central processes in cancer: stimulating angiogenesis, inhibiting apoptosis or modulating immune response [16]. In our study we identified several LPL species increased in high F_b_ EC patient tissues. Specifically, in this work we describe, for the first time, the potential role of lysophophatydilcholine (18:1), lysophosphatydilserine (14:0), lysophosphatydilinositol (20:0) and lysophosphatydilcholine (20:3) in EC tumour progression. These results reinforce the idea of LPL metabolism as a disease modifier in EC progression. Furthermore, this study has revealed that tissue resolvin D could be a good predictor of high tumour F_b_ (generally correlated to better patient survival). Resolvin D is known to be involved in the resolution of inflammation processes and shows potent effects in protecting against neovascularization and hypoxia in some pathological diseases [17]. Thus, our findings suggest that loss of the protective and inflammatory-resolution effect induced by tumour LPL and Resolvin D coexists with vascular proliferation leading to disorganized angiogenesis and hypoxia, relevant mechanisms in tumour progression and metastatic spread in EC [13].

All in all, the present work delves into the different signalling molecules involved in tumour progression. These achievements can serve to better understand EC metabolism helping to develop, in a near future, novel and more personalized therapies. Moreover, our results support the promising role of DCE-MRI as preoperative non-invasive imaging tool depicting tumour vascular features relevant for the corresponding metabolomic tumour signature that may be targetable in EC therapy.

## MATERIALS AND METHODS

### Patients and tissue samples

Endometrial tissue from hysterectomy specimens was collected from 26 patients surgically treated for endometrial carcinoma. All patients were diagnosed and treated at the same university hospital (Haukeland University Hospital, Bergen, Norway) written informed consent was obtained from all patients for the collection of imaging data and specimens for biomarker studies included in an institutional review board-approved protocol (Rek Vest 2009/2315).

### Dynamic contrast enhanced (DCE)-MRI protocol and blood flow (F_b_) analysis

Preoperative pelvic magnetic resonance imaging (MRI) was performed in all patients on a 1.5 Tesla MRI scanner (Siemens Avanto running Syngo v. B17, Germany) using a six-channel body coil and a standardized imaging protocol [6, 9] 20 mg butyl-scopolamine bromide (Buscopan; Boehringer, Germany) was administered intravenously prior to scanning in order to reduce motion artefacts. Contrast agent was administrated intravenously (Dotarem, Guerbet: 0.1 mmol gadolinium/kg, 3 ml/s injection speed). Pelvic sagittal and axial oblique (perpendicular to the long axis of the uterus) T2-weighted images were acquired together with axial oblique T1-weighted gradient-echo images prior to contrast agent administration. Pelvic DCE-MRI was obtained for 12 axial oblique slices applying a 3D spoiled gradient echo (FLASH) sequence (echo time/repetition time (TE/TR) = 1.05/2.64 ms, flip angle (FA) = 12°, matrix = 256x256, field of view (FOV) = 300 x 300 mm^2^, slice thickness = 5 mm, number of averages (NA) = 1). The temporal resolution was 2.49 s. and sequential images were acquired from 30 s before i.v. administration of contrast medium to 6.3 min after contrast injection. At 2 min post contrast medium administration, a pause of 33 s was utilized to acquire axial oblique T1-weighted gradient-echo contrast-enhanced images.

**Figure 4:**
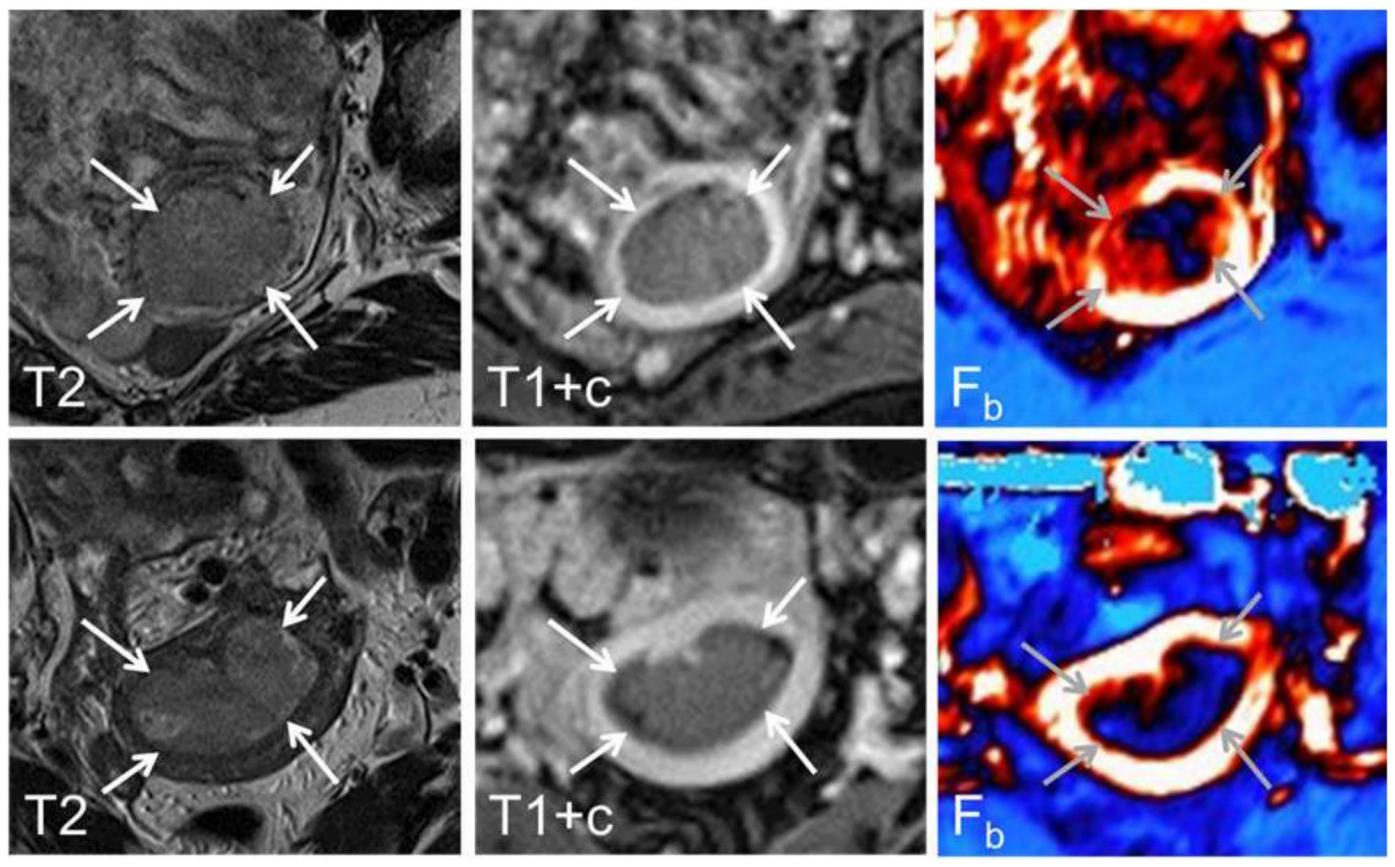
Axial oblique T2-weighted MRI (left panel), contrast-enhanced T1-weighted MRI (middle panel) and parametric blood flow (F_b_, right panel) map from a 68 year-old patient with FIGO stage 1B, grade 3 and high tumour F_b_ (upper panel) and a 52 year-old patient with FIGO stage 1B, grade 1 and low tumour F_b_ (lower panel). At conventional diagnostic MRI the endometrial carcinomas (arrows) are typically depicted as hyper-intense relative to the surrounding myometrium on T2-weigthed image while hypo-intense on contrast-enhanced T1-weighed image. On the corresponding parametric maps high blood flow is depicted as white/red while low blood flow is depicted as dark blue.

Regions of interests (ROIs) were drawn within the endometrial tumour by a radiologist (ISH with > 7 years of experience with pelvic MRI) who was blinded for tumour stage, histological diagnosis and patient outcome. Care was taken to avoid including necrotic and haemorrhagic areas of the tumours in the ROIs. The dynamic image series were analysed using an in-house implementation of the adiabatic approximation model of Johnson and Wilson [18]. Representative diagnostic MRI images and parametric DCE-MRI maps illustrating endometrial tumours with high and low blood flow (F_b_) are shown in Figure 4.

### Metabolomic analyses

Tissue samples (50–70 mg) were homogenized using 20 volumes of cold methanol as previously described [14]. Metabolite extracts were subjected to mass spectrometry using an HPLC 1290 series coupled to an ESI-Q-TOF MS/MS 6520 (Agilent Technologies, Santa Clara, CA, USA) as previously described [14]. Multivariate statistic analyses were performed using Metaboanalyst platform [19]. The preliminary identification of differential metabolites (Student’s T-Test, Benjamini Hochberg False Discovery Rate, p<0.05) was performed using the PCDL database from Agilent (Agilent Technologies, Barcelona, Spain), which accounts retention times in a standardized chromatographic system, exact mass and isotope distribution as an orthogonal searchable parameters to complement accurate mass data (AMRT approach) according to previously published works [14]. The version of the PCDL database used had retention times and accurate mass data for 679 compounds. To complete the identification process we searched for unidentified metabolites in Metlin Database (https://metlin.scripps.edu/index.php) which includes accurate masses and MS/MS spectrum for 961.829 molecules.

### Ethical approval

All procedures performed in studies involving human participants were in accordance with the ethical standards of the institutional research committee and with the 1964 Declaration of Helsinki and its later amendments or comparable ethical standards. Written informed consent was obtained from all patients for the collection of imaging data and specimens for biomarker studies; included in an institutional review board-approved protocol (Rek Vest 2009/2315). This article does not contain any studies with animals performed by any of the authors.

## SUPPLEMENTARY MATERIALS FIGURES



## Abbreviations

EC: Endometrial Cancer
DCE-MRI: Dynamic Contrast-Enhanced Magnetic Resonance Imaging
Fb: Blood Flow
FIGO: Federation International of Gynecology and Obstetrics
LPL: Lysophospholipids
RF: Random Forest
ROC: Receiver operating characteristics
ROI: Region of Interest
PCA: Principal Component Analysis
PLS-DA: Partial Least Discriminate Analysis
