# Supplementary material for: Tumour-microenvironmental blood flow determines a metabolomic signature identifying lysophospholipids and resolvin D as biomarkers in endometrial cancer patients
Source: Oncotarget. 2017 Nov 20;8(65):109018–26. doi: 10.18632/oncotarget.22558 (PMC5752500; doi:10.18632/oncotarget.22558)
Supplement: Supplementary file 1 [file oncotarget-08-109018-s001.pdf]

Tumour-microenvironmental blood flow determines a metabolomic signature identifying lysophospholipids and resolvin D as biomarkers in endometrial cancer patients

SUPPLEMENTARY MATERIALS

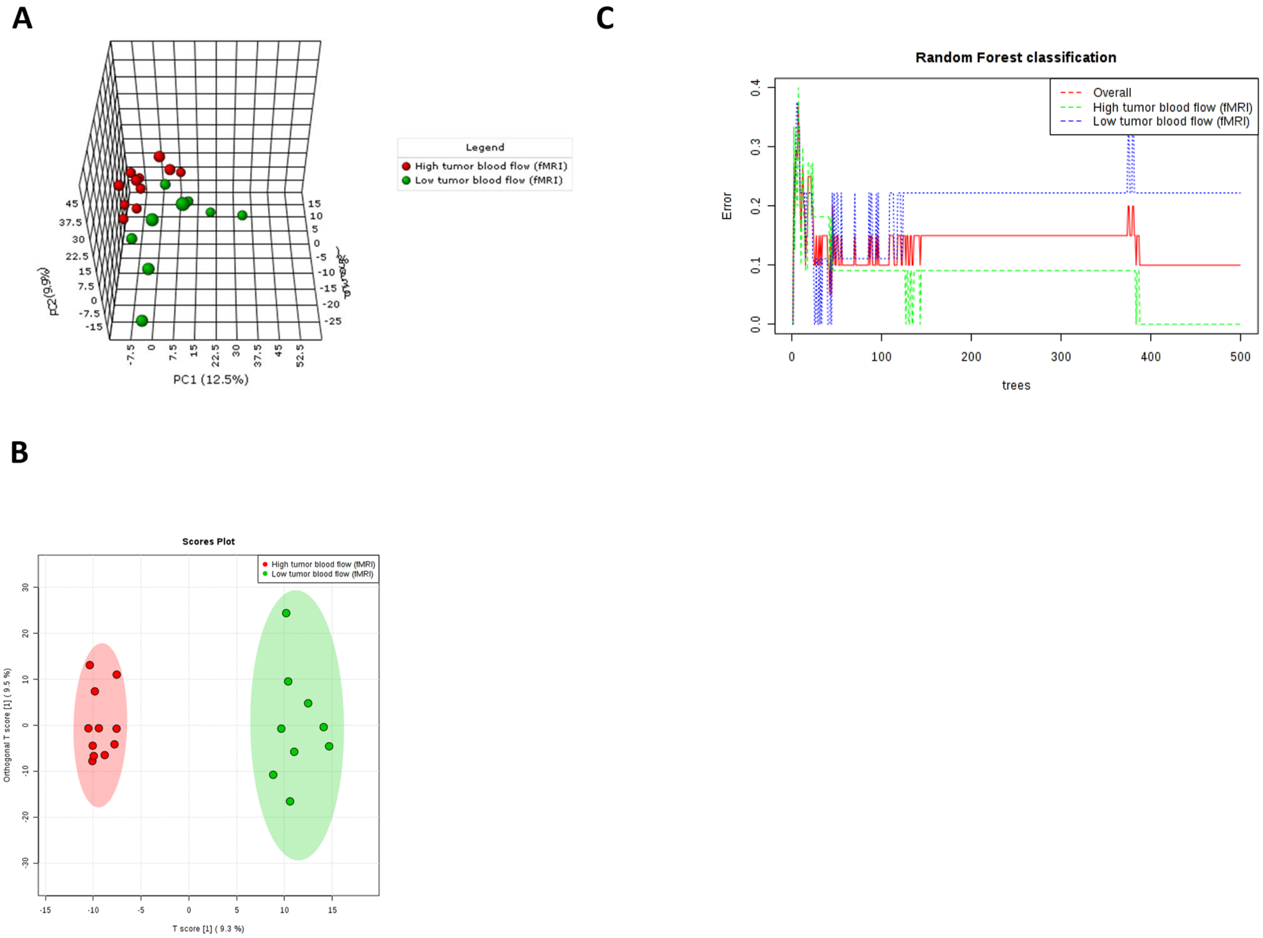

Supplementary Figure 1: (A) PCA demonstrates a metabolomic-specific signature depending on tumour blood flow in endometroid endometrial cancer patients. **(B)** Ortho-PLSDA algorithm using all metabolites found in samples from endometriod endometrial tumours is able to discriminate groups with an observed Q2 of 0.553 and observed R2Y of 0.979. Red spots represent high tumour blood flow patients and green spots represent low tumour blood flow as determined by DCE- MRI. **(C)** RF classification of endometroid endometrial cancer patients shows and overall classification error of 0.1.

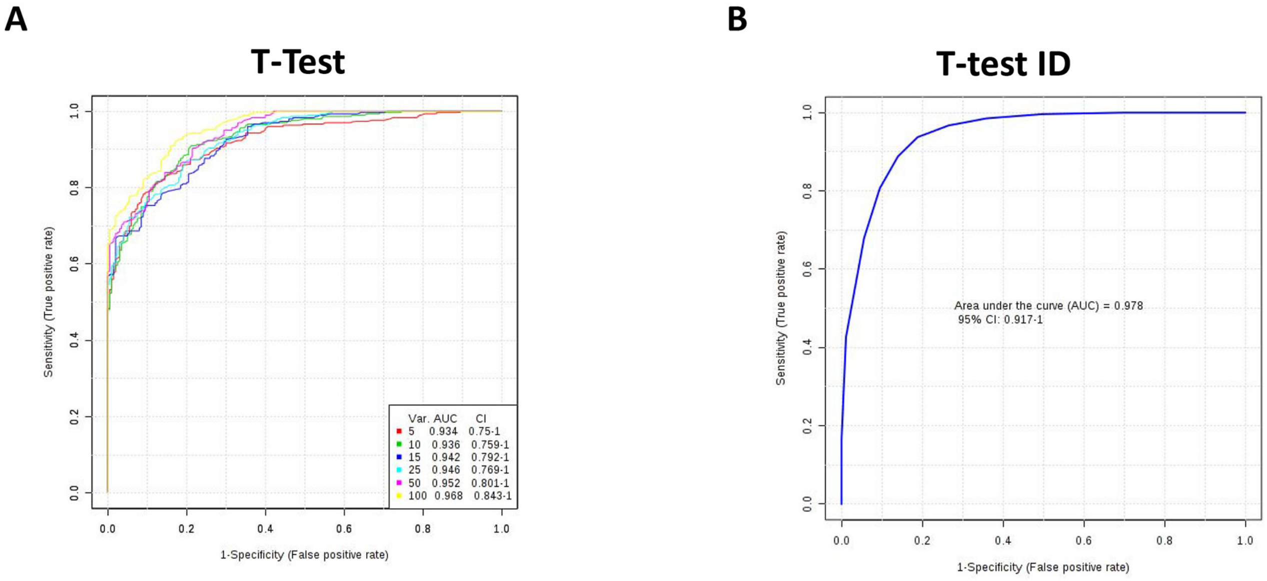

Supplementary Figure 2: ROC curves using groups of metabolites (5, 10, 15, 25, 50 and 100) with lowest p-value obtained from T-Test between endometrial cancer tumours with high versus low blood flow **(A)** and using only the significant ones (T-Test, Benjamini Hochberg False Discovery Rate, p<0. 05) with a potential identity **(B)**.
